# Enhancing the Topical Antibacterial Activity of Fusidic Acid via Embedding into Cinnamon Oil Nano-Lipid Carrier

**DOI:** 10.3390/gels10040268

**Published:** 2024-04-16

**Authors:** Heba S. Elsewedy, Tamer M. Shehata, Shaymaa M. Genedy, Khuzama M. Siddiq, Bushra Y. Asiri, Rehab A. Alshammari, Sarah I. Bukhari, Adeola T. Kola-Mustapha, Heba A. Ramadan, Wafaa E. Soliman

**Affiliations:** 1 Department of Pharmaceutical Sciences, College of Pharmacy, AlMaarefa University, Diriyah 13713, Saudi Arabia; 2 Department of Pharmaceutical Sciences, College of Clinical Pharmacy, King Faisal University, Alhofuf 36362, Saudi Arabia; 3 Department of Pharmaceutics, College of Pharmacy, Zagazig University, Zagazig 44519, Egypt; 4 Department of Pharmaceutics, College of Pharmacy, King Saud University, Riyadh 11451, Saudi Arabia; 5 Department of Pharmaceutical Sciences, College of Pharmacy, Alfaisal University, Riyadh 11533, Saudi Arabia; 6 Department of Pharmaceutics and Industrial Pharmacy, Faculty of Pharmaceutical Sciences, University of Ilorin, Ilorin 240003, Nigeria; 7 Department of Microbiology and Immunology, Faculty of Pharmacy, Delta University for Science and Technology, Gamasa, Mansoura 11152, Egypt; 8 Department of Biomedical Sciences, College of Clinical Pharmacy, King Faisal University, Alhofuf 36362, Saudi Arabia

**Keywords:** fusidic acid, cinnamon essential oil, nanoemulsion, optimization, topical delivery, antibacterial activity

## Abstract

Presently, antimicrobial resistance is of great risk to remarkable improvements in health conditions and infection management. Resistance to various antibiotics has been considered a great obstacle in their usage, necessitating alternative strategies for enhancing the antibacterial effect. Combination therapy has been recognized as a considerable strategy that could improve the therapeutic influence of antibacterial agents. Therefore, the aim of this study was to combine the antibacterial action of compounds of natural origin like fusidic acid (FA) and cinnamon essential oil (CEO) for synergistic effects. A distinctive nanoemulsion (NE) was developed using cinnamon oil loaded with FA. Applying the Box–Behnken design (BBD) approach, one optimized formula was selected and integrated into a gel base to provide an FA-NE-hydrogel for optimal topical application. The FA-NE-hydrogel was examined physically, studied for in vitro release, and investigated for stability upon storage at different conditions, at room (25 °C) and refrigerator (4 °C) temperatures, for up to 3 months. Ultimately, the NE-hydrogel preparation was inspected for its antibacterial behavior using multidrug-resistant bacteria and checked by scanning electron microscopy. The FA-NE-hydrogel formulation demonstrated a pH (6.32), viscosity (12,680 cP), and spreadability (56.7 mm) that are acceptable for topical application. The in vitro release could be extended for 6 h, providing 52.0%. The formulation was stable under both test conditions for up to 3 months of storage. Finally, the FA-NE-hydrogel was found to inhibit the bacterial growth of not only Gram-positive but also Gram-negative bacteria. The inhibition was further elucidated by a scanning electron micrograph, indicating the efficiency of CEO in enhancing the antibacterial influence of FA when combined in an NE system.

## 1. Introduction

Topical drug delivery systems are a quick and efficient method for transporting medication to a specific site over the skin [1]. They are an alternative to oral drug administration; thus, topical drug delivery could be a helpful technique, especially when the patient is a child or suffers from difficulty in swallowing [2]. Moreover, it can minimize the risk of gastrointestinal problems since it does not provide systemic absorption for the medication and avoids first-pass metabolism [3]. Topical drug delivery shows selectivity in delivering medication to specific affected areas. Further, patients demonstrate better compliance with such delivery owing to convenience, ease of application, and limited frequency of drug application [4]. A wide variety of drugs could be delivered via the topical route, providing anti-inflammatory [5], analgesic [6], anticancer [7], antifungal [8], wound healing [9], and antibacterial activity [10]. While this route of administration offers various advantages, it shows certain challenges as well. The particle size of a topical formulation plays a crucial role in its absorption through the skin [11]. In order to overcome such a problem and to maximize the efficacy of the contained pharmaceutical active agent, it requires its incorporation into a nanocarrier system exploiting the concept of nanotechnology [12].

Nanotechnology is an approach that has been broadly applied in several aspects, especially in the medical field [13]. The manipulation of nanotechnology in the medical field is based on producing smaller and more efficient formulations [14]. The aforementioned strategy is accomplished by developing certain nanocarriers with a tiny particle size and, hence, a large surface area, which could consequently improve the solubility and bioavailability of drugs [15]. Various nanocarriers have been manufactured; nevertheless, nano-lipid formulations are regarded as promising carriers in delivering drugs topically [16]. The benefit behind such nano-lipids is associated with their high stability, compatibility, low degradability, and ability to overcome the skin barrier [17,18]. Nano-lipid formulations comprise niosomes, liposomes, ethosomes, nanoemulsions (NEs), and more. It is worth mentioning that NEs exhibit more advantages over other nanocarriers owing to their small droplet size, which prevents sedimentation and provides higher stability [19]. Additionally, NEs are considered a proper system for delivering active agents through the skin owing to their safety since they are non-toxic and non-irritant. Moreover, the large surface area of NEs facilitates the penetration of drugs when applied over the skin [20]. NEs are believed to be good example of nanocarriers alongside other carriers that show promise and stability as topical nanomedicines [21]. Various pharmaceutical active agents could be entrapped within NEs to achieve different effects, including anti-inflammatory [22], analgesic, hypolipidemic [23], anticancer [24], and antibacterial effects [25].

Antibiotics are substances that work by killing or inhibiting the growth of bacteria; thus, they are so-called antibacterial drugs [26]. They are widely used for the treatment of various disorders caused by bacterial infection. Fusidic acid (FA), an antibacterial medication, is obtained naturally from the fungus Fusidium coccineum and has a tetracyclic triterpenoid structure. It was revealed that its mode of action in inhibiting bacterial growth is by attaching itself to bacterial ribosomes and preventing peptide translocation. Consequently, it would prevent the synthesis of proteins and causes the ribosomes to disassemble [27]. FA is available in a number of conventional dosage forms; however, skin infections require topical therapy most preferably, owing to its ability to target the affected area directly [28]. FA is classified as a class II drug based on the biopharmaceutical classification system, which means that it possesses high permeability and low solubility. Unfortunately, the hydrophobic nature of FA presents a great obstacle in its incorporation into pharmaceutical preparations, resulting in less therapeutic efficacy [29]. Moreover, antimicrobial resistance remains a major challenge standing as a hurdle in treating infectious diseases [30]. Since bacterial resistance to antibiotics mainly results from widespread misuse and inadequate monitoring of antibiotic administration [31], finding a treatment strategy to decrease microbial resistance is essential. 

Microbial resistance could be managed either by developing novel antibacterial medications or by increasing the effectiveness of already-approved drugs [32]. Using the nanotechnology approach for developing novel antibacterial medications could enhance the solubility and efficacy of the medication and provide a sustained release action that would result in lowering the frequency of application [29]. Moreover, using a combination therapy could accomplish great improvement in the antibacterial activity of certain antibiotics and increase their effectiveness [33]. In combinational therapy, antibiotics could be used with other substances that behave like antibacterial agents. In recent times, a great attention has been paid to the activity of essential oils as natural compounds in monitoring several disorders [34]. Using these essential oils heavily relies on their availability as natural compounds, exhibiting safety and efficacy in their utilization, provided they are used in a proper way [35]. One essential oil, namely cinnamon essential oil (CEO), has exhibited antibacterial activity against both Gram-positive and Gram-negative bacteria [36]. It is derived from the Cinnamomum plant that belongs to the Lauraceae family. The most important constituents of CEO are cinnamaldehyde and eugenol, in addition to other components like eucalyptol, camphor, and Linalool [37]. Moreover, it has been established that, in addition, CEO exhibits anti-inflammatory, antifungal, and antihypertensive effects [38]. 

Considering the aforementioned facts, the current study is an attempt to improve the antibacterial efficacy of FA to be active against different bacterial strains by exploiting combination therapy. Therefore, FA was incorporated into an NE formulation prepared with CEO by applying the quality by design approach to obtain a high-quality product, and selecting the optimized NE formulation. As far as we know, this is the first combination therapy using FA and CEO. The optimized formula was integrated with a hydrogel base to be applied evenly over the skin. The developed FA-NE-hydrogel was characterized as a topical formulation, examining the pH, viscosity, and spreadability of the formulation. An in vitro release kinetic study was also performed in addition to a stability study. Further, the formulation was inspected for its antibacterial activity and the bacteria were checked for their morphology after being treated with the developed formulation.

## 2. Results and Discussion

### 2.1. Model Fitting and Design Validation

Referring to Table 1, 15 formulations were organized by BBD and developed depending on definite factors and their influence on the observed responses. An ANOVA in BBD software (version 12.0) provided a statistical analysis for all observed responses, which is very necessary for fitting the design model as displayed in Table 2. It was obvious that the model F-values for both responses were 260.50 and 288.66 for R_1_ and R_2_, respectively, which indicates a significant model. Furthermore, *p*-values were found to be significant for most of the model terms given that their values were less than 0.05. Another essential parameter for model fitting is the lack of fit value since it is highly recommended to have non-significant lack of fit. In the obtained results, the lack of fit values were 2.89 and 0.0833 with corresponding *p*-values of 0.2673 and 0.9630 for R_1_ and R_2_, respectively, which suggests non-significant lack of fit [32]. 

### 2.2. Analysis of the Detected Response

#### 2.2.1. Effect of Selected Factors on Particle Size

The effects of selected factors on particle size were measured, and their values were recorded between 209 ± 2.6 and 308 ± 4.8 nm, with corresponding PDI values of 0.237 ± 0.022 and 0.402 ± 0.011 for F3 and F5, respectively. It was revealed that the particle size distribution of all NE formulations were in a narrow range of sizes, which is a worthy sign of the good stability of the formulation [39]. As per the data, the three selected factors exhibited a great influence based on the formulation particle size. Regarding factor A, related to the CEO concentration, it was noted that increasing factor A would result in a parallel increase in particle size as well. The reason behind this could be the aggregation and coalescence that are proposed to happen upon using higher oil concentrations. Once the particles aggregated together, it would provide a larger particle size, adding to the possible increase in the dispersed phase [40]. On the contrary, using a higher concentration of tween 80, factor B, would diminish the formulation’s particle size while using same concentration of CEO. This is attributable to the key role of a surfactant that reduces the interfacial tension between the oil phase and aqueous phase [41]. Furthermore, a surfactant could cover the particle-forming layer and might be a hindrance to particle aggregation [42]. On the same track, using a higher concentration of factor C, Transcutol^®^ P, while keeping the same concentration of factors A and B would result in a smaller particle size. Similarly, this finding could be ascribed to the previous fact regarding the surfactant and co-surfactant’s role in decreasing interfacial tension, which would decrease the particle size of the formulation consequently. 

The obtained results could be simply confirmed by a mathematical equation generated from the design software. As is clear, the positive sign behind factor A emphasizes the parallel relationship between that factor and the resulting response, R_1_. However, the negative sign in front of factors B and C points toward their antagonistic influence on the same studied response, R_1_

R_1_ = 245.667 + 35.25 × A − 12.375 × B − 3.875 × C − 6.25 × AB − 1.75 × AC − 3.65585e^−16^ × BC + 7.16667 × A^2^ + −0.583333 × B^2^ − 2.58333 × C^2^

**Figure 1:**
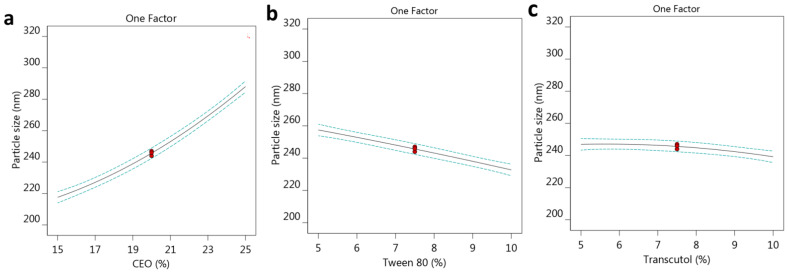
One factor graph showing the influence of different factors (**a**) CEO, (**b**) Tween 80, and (**c**) Transcutol^®^ P concentrations on the investigated particle size R_1_.

**Figure 2:**
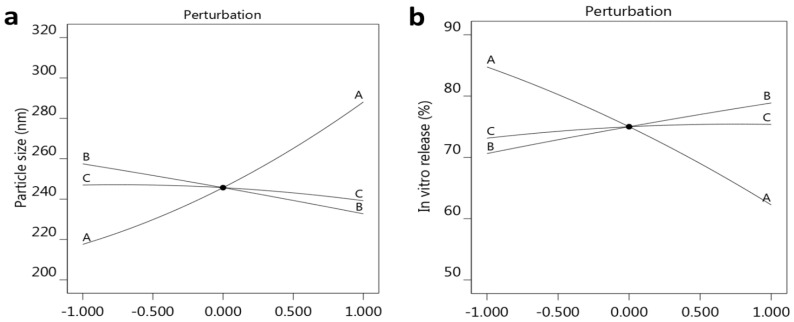
Perturbation plot displaying the influence of each factor (A, B, and C) alone on (**a**) particle size R_1_ and (**b**) in vitro release R_2_.

For more confirmation, the design software created some graphs that illustrated and confirmed the influence of the three selected factors on the observed response R_1_. As depicted in Figure 1a–c, one factor graph for each factor was plotted against the particle size response. It was apparent from the graph that the response (R_1_) related to the particle size of the developed FA-NE formulations would increase by increasing the concentration of factor A, whereas it would decrease upon increasing the other factors B and C. Furthermore, as seen in Figure 2a, which is referred to a perturbation plot, the most prominent effect was distinguished in factor A when compared to factors B and C. 

Additionally, a linear correlation between the predicted and the actual values of the response was apparent as displayed in Table 3, where both values of the predicted and adjusted R^2^ were very close to each other since the difference between them was less than 0.2. This indicates that they were in a reasonable agreement with each other. Moreover, the value of adequate precision seemed to be an adequate signal that navigates the design space.

#### 2.2.2. Effect of the Selected Factors on In Vitro Release

The in vitro release of FA from the developed NE formulations was assessed and found to range from 58 ± 2.5 to 89 ± 3.6%. It was remarkable that the selected factors distinctly showed a notable influence on response R_2_. As per the data shown in Table 1, it was observable that there was a marked reversible influence between factor A and response R_2_, which mean that upon increasing the CEO concentration, the in vitro release would markedly decrease. This is supposed to be due to the larger particle size that was obtained while using higher oil concentrations, which would provide a smaller surface area and, consequently, lower the percentage of drug released [43]. 

On the other hand, a direct relation was detected between factors B and C and the in vitro release response (R_2_). Subsequently, increasing the concentration of tween 80 and Transcutol would maximize the percentage of FA released from the NE formulation. This was owed to the small particle size that was obtained while using a higher concentration of factors B and C, since small particle sizes could provide larger surface areas, and a larger amount of the drug could be released as well. The previous data were further confirmed from the obtained mathematical equation, where factor A carried a negative sign, signifying an opposite effect. On the contrary, factors B and C possessed a positive sign, standing for a positive synergistic influence [44], as follows:

R_2_ = 75 − 11.25 × A + 4.125 × B + 1.125 × C − 0.25 × AB + 0.25 × AC + 1.17757e^−16^ × BC − 1.5 × A^2^ − 0.25 × B^2^ − 0.75 × C^2^

**Figure 3:**
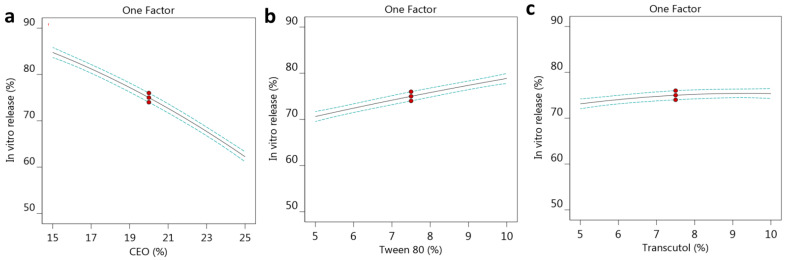
One factor graph screening the influence of factor (**a**) CEO, (**b**) Tween 80, and (**c**) Transcutol concentrations on the examined in vitro release (R_2_).

Likewise, the relation between the selected factors and the observed response are illustrated further by the representative graphs, depicted as one-factor graphs, shown in Figure 3a–c. It was obvious that the CEO concentration (A) exerted an opposite influence on the response, since increasing this factor (A) would result in a subsequent decrease in in vitro release. On the contrary, other factors, B and C, presented a direct correlation with the R_2_ response, where their increment exhibited a consequent rise in in vitro release. Additionally, Figure 2b, showing a perturbation plot, reveals that the most noticeable effect was represented by factor A, comparable to the other two factors, B and C. Regarding the correlation between the predicted and actual values of in vitro release, it was revealed to be a linear relation because there was a viable agreement between the predicted and adjusted R^2^, values since the difference between them was less than 0.2, as displayed in Table 3.

### 2.3. Verification of Optimization Process

**Figure 4:**
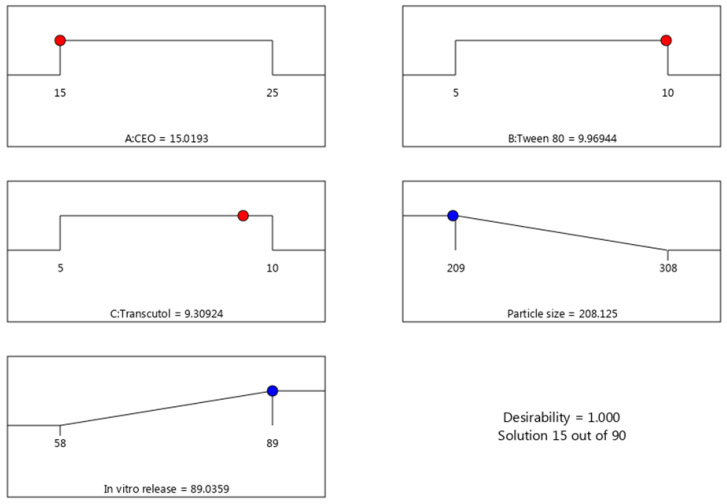
Optimization ramps for the examined factors a long with the predicted values of responses.

**Figure 5:**
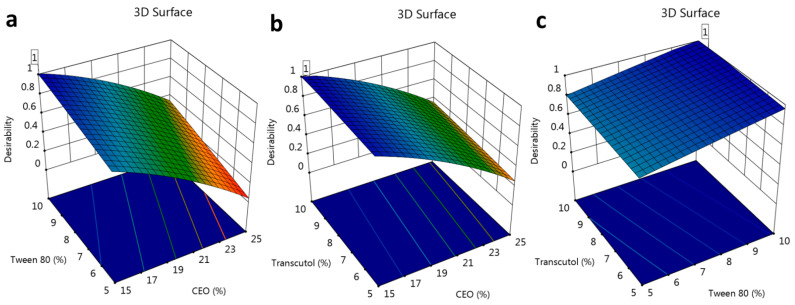
Three-dimensional surface optimization plot showing the effect of different factors (**a**) CEO and Tween 80, (**b**) CEO and Transcutol, and (**c**) Tween 80 and Transcutol on overall desirability.

**Figure 6:**
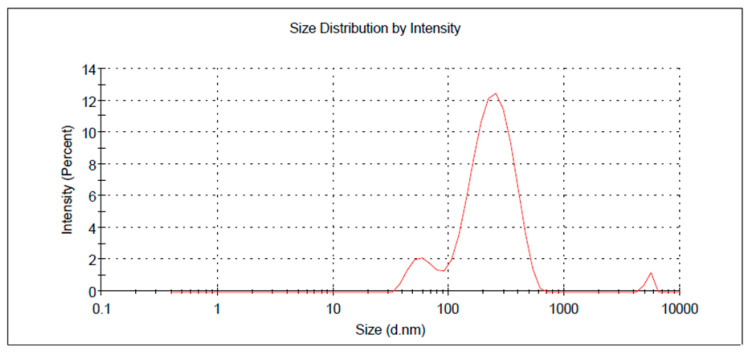
Particle size of optimized FA-NE formulation.

In order to optimize the developed formulations, numerical optimization was carried out. The responses were directed toward certain relevant standards to reach higher-quality formulations. In the current design, the factors were assigned within a certain range while the responses were fixed to minimize the particle size and to maximize the in vitro release. The report of the solution in the numerical optimization provided several assumptions. The highest desirability value was selected (1.000), proposing the recommended concentration of all factors, as displayed in Table 4 and Figure 4 and Figure 5. Based on that assumption, a new FA-NE formulation was developed to be the optimized formula after observing the considerable similarity between the predicted and the observed values. Figure 6 demonstrates the particle size of the optimized FA-NE formulation, which is 202.4 nm, with its PDI value of 0.314.

### 2.4. Compatibility Studies (Fourier Transform Infrared Spectroscopy (FTIR) Studies)

**Figure 7:**
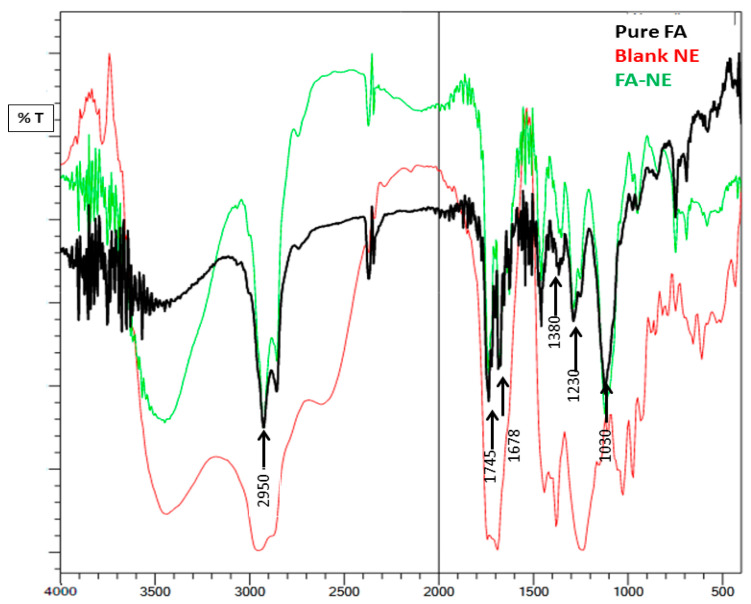
FTIR spectra representing pure FA, blank NE and optimized FA-NE formulation.

As shown in Figure 7, the spectra of pure FA, blank NE, and optimized FA-NE were analyzed to check for any interaction between the ingredients of the formulation. Distinct beaks related to FA were seen at 2950, 1380, 1230 cm^−1^, and 1030 cm^−1^. These peaks are related to C–H stretching, C–H bending, the C–O stretching of carbonyl groups, and aromatic C=C, respectively. Additionally, a double peak was seen in the spectrum at 1745 and 1678 cm^−1^, which is attributed to the C=O stretching vibrations of the acetyl and carbonyl groups, respectively. Our result was in agreement with those of Marian et al., 2020, who concluded similar peaks for fusidic acid at 2953, 1380,1686, 1742, 1260, and 1027 cm^−1^, which seem to be very close to our detected peaks [45]. On the other hand, when incorporated in a nanoemulsion, significant FA physical interactions were exhibited, as most of the characteristic peaks of FA were diffused in the FTIR spectra of the nanoemulsion, such as peaks at 1230 cm^−1^ and 1030 cm^−1^.

### 2.5. FA-NE-Hydrogel Characterization

According to the optimization process and its impact, the optimized NE preparation integrating FA was formulated and then incorporated into a pre-formulated HPMC gel base. This produced a smooth, homogenous, and stable NE-hydrogel without any sign of phase separation upon visual examination. Since it is important to determine whether a topical formulation is an irritant or not, the pH value of the hydrogel formulation was evaluated to be 6.32 ± 0.26, which is very close to the pH of skin, suggesting the safety of the formulation upon topical application [46]. Regarding the viscosity of topical formulations, it is a vital requirement to be evaluated as it influences the rate of drug diffusion from the formulation and disturbs the behavior of in vitro release [47]. Accordingly, the viscosity of the FA-NE-hydrogel was evaluated and found to be 12,680 ± 1045 cP, which is within the acceptable limit for topical application. Relating to the spreadability of topical preparations, it is important to identify how easily the formulation would spread over the affected area when topically applied. Patient convenience is achieved when a formulation spreads easily and consistently. The spreadability of the examined FA-NE-hydrogel formulation was detected to be 56.7 ± 1.5 mm [41]. 

### 2.6. In Vitro Release from NE-Hydrogel Formulation

**Figure 8:**
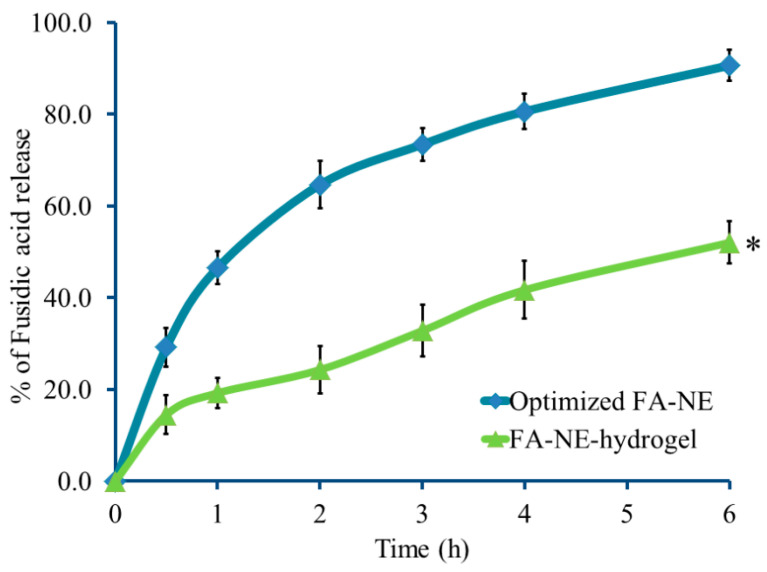
In vitro release study of FA from NE-hydrogel compared to optimized NE formulation over 6 h. Results are expressed as mean value ± SD, (n = 3). * *p* < 0.05 compared with optimized FA-NE formulation.

FA’s release from the NE-hydrogel was assessed and compared to that released from an optimized NE, as portrayed in Figure 8. As validated from BBD, the percentage of FA released from the optimized NE reached 90.66 ± 3.43% over 6 h; however, it was 52.0 ± 4.6% when released from the NE-hydrogel formulation. In fact, the percentage of FA released from the NE was found to be significantly greater than that released from the NE-hydrogel formulation (*p* < 0.05). This might be attributable to presence of FA in the external aqueous phase of the NE formulation, which results in an easier and larger release of the drug to the media. On the other hand, the release from the NE-hydrogel was much delayed as a result of the inclusion of the drug into the NE and then into a gel base preparation, a system that would offer more layers for the drug to be released out of the carrier. In addition, the higher viscosity of the gel base formula would slow down the rate of drug release from the formulation [48].

### 2.7. Kinetic Study

Various kinetic models were investigated to detect the distinct process by which FA was released from NE and NEG formulations. A curve was created to illustrate the relation between drug release and time, and the most linear plot with the highest R^2^ value was identified. Consequently, the investigation demonstrated that FA release from both formulations complied with Higuchi kinetic models. When compared to other investigated kinetic models, the Higuchi model offers the highest linear correlation and the highest R^2^ value: 0.9772 and 0.9843 for optimized FA-NE and FA-NEG, respectively, as shown in Table 5. It is commonly known that the release of a drug is supposed to follow the Higuchi kinetic model when it diffuses from its lipid matrix under controlled conditions [49]. Furthermore, it is renowned that the best model for characterizing the dissolution of medication in case of topical formulations is the Higuchi model [50].

### 2.8. Stability Test

**Figure 9:**
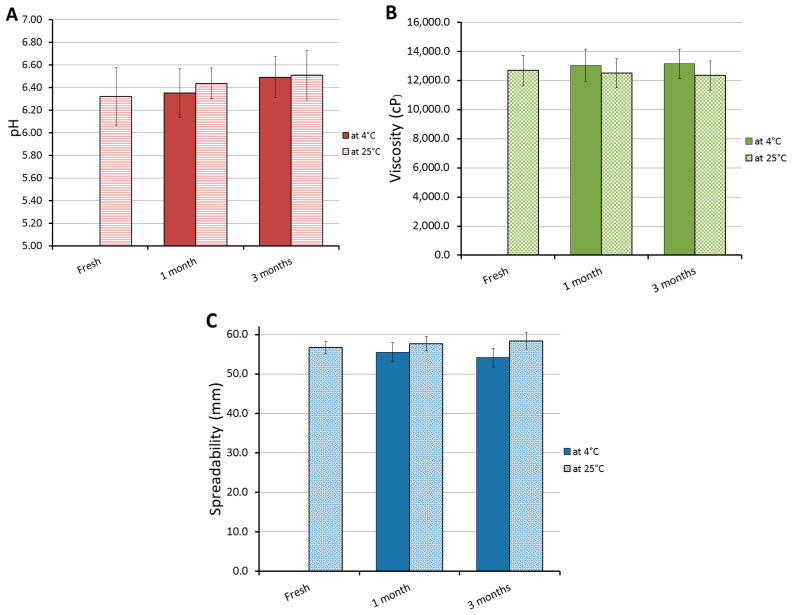
Stability of FA-NE-hydrogel following 1 and 3 months of storage at 25 ± 2 °C and at 4 ± 3 °C relative to (**A**) pH, (**B**) viscosity, and (**C**) spreadability compared to fresh formulation. Data are expressed as mean ± SD for three experiments.

The physical stability of the formulated FA-NE-hydrogel formulation was checked by investigating certain parameters that were previously studied for the fresh formulation, such as pH, viscosity, spreadability, and in vitro release. The formulation was stored in two different conditions—25 ± 2 °C and 4 ± 3 °C for a period of 1 and 3 months—as explained in Figure 9. The results exhibited revealed that the difference was non-significant in all parameters for the formulation after being stored in both conditions for up to 3 months when compared to the fresh formula (* *p* < 0.05). The previous claim that NE formulas are very stable was further substantiated. This is due to NEs’ composition and small particle size, which strongly influence the stability behavior of the dosage form during storage [51].

### 2.9. In Vivo Study: Skin Irritation Test

An animal back treated with the FA-NE-hydrogel formulation was checked for any reaction that might have happened. It was noticed that no inflammation, irritation, erythema, or edema was observed on the animal back skin during the whole 7 days of the study, which points to the relative safety of the formulations.

### 2.10. Antibacterial Study

To detect the efficiency of FA and cinnamon oil against various bacterial strains, an antibacterial study was carried out. Simply, the antibacterial behavior was recognized by measuring the bacterial inhibition zone caused by the formulation under investigation. As per the data in Table 6 and Figure S1, different formulations, including FA-NE-hydrogel, blank NE, and FA marketed products, exhibited inhibition zones against different bacteria. It was highly obvious that the FA-NE-hydrogel formulation caused significant inhibition for *Staphylococcus aureus* and *E-coli* when compared to the blank NE-hydrogel (*p* < 0.05), which indicates its effectiveness against these bacteria. Interestingly, remarkable bacterial inhibition was observed by the blank NE-hydrogel, which was ascribed to the role of CEO in inhibiting the bacterial growth. It was previously reported that CEO possesses significant antibacterial action owing to the activity of its major component, transcinnamaldehyde [52,53]. Previous studies confirm the antibacterial activity of CEO against a wide range of Gram-positive and Gram-negative bacteria [54]. With regard to the former consequences, the higher antibacterial activity offered by the FA-NE-hydrogel formula could be credited to the combination of FA with cinnamon oil that gave rise to antibacterial synergism. It is noteworthy that such a combination provided greater antibacterial action against Gram-positive as well as Gram-negative bacteria, although it has been stated previously that FA alone possesses limited effectiveness against Gram-negative bacteria [55]. This result confirms the effectiveness of CEO and combination therapy as a good strategy to improve the efficacy of the drug and overcome bacterial resistance. Interestingly, several studies tried to investigate the synergistic action between different components in order to enhance the efficacy [56].

**Figure 10:**
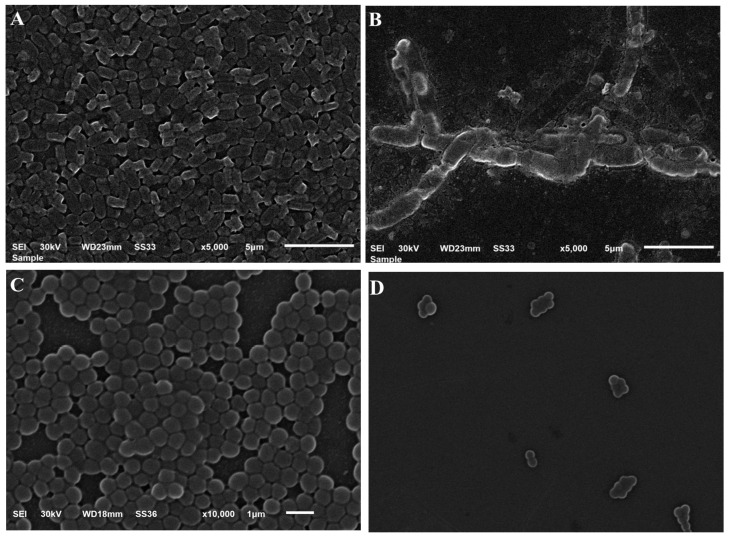
SEM screening of the morphology of (**A**) control *Staphylococcus aureus*; (**B**) *Staphylococcus aureus* treated with FA-NE-hydrogel, (**C**) control *E-coli*, and (**D**) *E-coli* treated with FA-NE-hydrogel.

Antibacterial and anti-biofilm characteristics were investigated using SEM, as presented in Figure 10. It was obvious that there was a reduction in the bacterial cell number in addition to visible alterations and irregularities in the cell structure after treating with the FA-NE-hydrogel comparable with the control sample. These observations suggest that bacterial damage occurred, including bacterial cell wall destruction, that might have led to cell death.

## 3. Conclusions

The present study is an attempt to overcome the resistance of certain bacterial species to antibacterial drugs. This study aimed to apply a combination therapy strategy where fusidic acid was loaded into a nanoemulsion formulation prepared with cinnamon oil. The quality by design approach was functionally deployed to develop various nanoemulsion formulations depending on selected factors and their consequence on the studied response. For more convenient topical application, the optimized formula, with a particle size of 202.4 nm and an in vitro release of 90.66%, was integrated into a gel base to obtain an FA-NE-hydrogel formulation. The FA-NE-hydrogel showed good physical characteristics, with a pH (6.32), viscosity (12,680 cP), and spreadability (56.7 mm) that were adequate for topical application. The kinetic study showed that Higuchi was the best-fit model with an r^2^ value of 0.9843. The formula was proven to be stable upon storage at room temperature and under refrigeration for a period of 3 months. Finally, the antibacterial activity of FA was significantly enhanced by cinnamon oil, which has potential for providing better antibacterial effects against a wide variety of bacteria. 

## 4. Materials and Methods

### 4.1. Materials

FA was brought from the Saudi Pharmaceutical Industries & Medical Appliances Corporation (SPIMACO ADDWAEIH, Buraydah, Saudi Arabia). CEO was bought from AVD Organics (Veena Industrial Estate, LBS Marg, Vikhroli West, Mumbai, India). Diethylene Glycol Monoethyl Ether (Transcutol^®^ P) was procured from Gattefosse SAS (Lyon, France). Tween 80 was acquired from ALPHA CHEMIKA (Mumbai, India). The gelling agent, Hydroxypropyl methylcellulose (HPMC) was purchased from Sigma-Aldrich Co. (St. Louis, MO, USA). All other chemicals and solvents were of analytical grade.

### 4.2. Box–Behnken Experimental Design (BBD)

BBD version 12 software, a response surface methodology approach, was employed using Design-Expert, version 12.0 (Stat-Ease, Minneapolis, MN, USA), to obtain high-quality formulations. Based on that, three-factor, three-level (33) factorial design was used to develop 15 different runs. Certain factors were selected, namely the oil, surfactant, and co-surfactant concentration, representing A, B, and C, respectively. These factors were studied for their influence on some responses. The investigated responses were the particle size and percentage of in vitro release, R_1_ and R_2_, respectively, as displayed in Table 7. The obtained data were analyzed statistically using an analysis of variance (ANOVA), producing mathematical equations that clarify the relation between the studied factors and the obtained response. Furthermore, certain graphs were exported from the design software for extra elucidation.

### 4.3. Developing FA-NE

Different FA-NE formulations were developed using the specified concentration proposed by BBD for each factor, as presented in Table 1. CEO was used to form the oily phase; tween 80 is an excellent surfactant, in addition to Transcutol, which is an excellent co-surfactant and solubilizer. Two phases, an oily and aqueous phase, had to be prepared. For the oily phase, 50 mg of FA was dissolved in CEO (15–20%) and mixed with Transcutol (5–10%) as a co-surfactant using a classic advanced vortex mixer (VELP Scintifica, Rome, Italy). The aqueous phase, up to 10 mL, was prepared as distilled water emulsified with tween 80 as a surfactant (5–10%). Next, the aqueous phase was gradually added to the oily phase while homogenizing using a high-shear homogenizer (T 25 digital Ultra-Turrax, IKA, Staufen, Germany) until the emulsion was formed. The developed emulsion was sonicated for 30 s using the probe sonicator XL-200, Qsnonica (New town, CT, USA), to attain a suitable particle size [56]. 

### 4.4. Particle Size and Size Distribution (PDI)

About 5 µL of each formulated FA-NE was added to a disposable cuvette to be diluted with 3 mL of distilled water and then assessed for its particle size and polydispersity index (PDI). This evaluation was performed using the dynamic light scattering technique via a Malvern Zetasizer (Nanoseries, zs; Malvern Instruments, Malvern, UK) at 25 °C [57].

### 4.5. In Vitro Release Study

To determine the concentration of FA released from all NE formulations, the ERWEKA dissolution system (ERWEKA, GmbH, Heusenstamm, Germany) was operated. A sample of FA-NE was placed onto a cover fixed to a glass tube, wrapped with a cellophane membrane (MWCO 2000–15,000), and attached to the apparatus. The glass tubes were suspended into the acceptor media, which consisted of 500 mL of phosphate buffer (pH 5.5), adjusted at 32 ± 0.5 °C, and allowed to rotate at 50 rpm. After running the experiment, a sample of 3 mL was withdrawn from the media to be analyzed spectrophotometrically at λmax 285 nm using a UV Spectrophotometer (JENWAY 6305, Bibby Scientific Ltd., Staffs, UK). The withdrawn sample, at various time intervals, was replaced with an equivalent amount of fresh media [58]. Each investigation was performed at least three times with the mean value ± SD. 

### 4.6. Compatibility Studies (Fourier—Transform Infrared Spectroscopy (FTIR) Studies)

The probable interaction between the drug and other ingredients included in the formulation can be estimated via FTIR study. The study was performed using an FTIR spectrophotometer (FTIR spectrophotometer, SHIMADZU, IRAFFINITY-1S, Tokyo, Japan) to apply the KBr pellet method. The examined preparations were the pure drug or the developed formulation. A KBr plate was prepared by compressing KBr with the pure drug, while for the developed formulation, the KBr plate was compressed and a small amount of the examined formulation was applied over it. The study was performed using spectra between 4000 and 400 cm^−1^, measured at a resolution of 8 cm^−1^ with 40 scans [59].

### 4.7. Developing FA-NE-Hydrogel

For the proper application of a topical formulation over the skin, a viscous preparation is preferable. In pursuit of that goal, the optimized FA-NE formulation was integrated into a pre-formulated gel preparation. HPMC was used since it is a good gelling agent, resulting in forming a homogenous and consistent hydrogel with good viscosity and stability [60]. In essence, a 4% gelling agent, mainly HPMC, was dispersed gently over 10 mL of distilled water, and the mixture was stirred using a magnetic stirrer (Jeio Tech TM-14SB, Medline Scientific, Oxfordshire, UK) until the gel base was prepared [59,61]. Optimized FA-NE was added to the pre-prepared hydrogel base while mixing for 10 min using a mixer (Heidolph RZR 1, Heidolph Instruments, Schwabach, Germany) until homogenous FA-NE-hydrogel was acquired.

### 4.8. FA-NE-Hydrogel Characterization

Different characterization tools were utilized to analyze the formulated gel preparation [10]. Organoleptic examination was the first evaluation performed on the FA-NE-hydrogel, where the preparation was checked visually for its appearance, homogeneity, and consistency. Further, the pH of a topical formula is highly considered for assuring its safety when applied over the skin. Evaluating the pH value was performed using a pH meter (MW802, Milwaukee Instruments, Szeged, Hungary). The viscosity of the formulation was another parameter to be evaluated. This was performed using a Brookfield viscometer (DV-II+ Pro, Middleboro, MA, USA) at 25 °C, rotating at 0.5 rpm using spindle R5 [24]. Moreover, the ability of the formulation to be spread consistently upon application over the skin was also estimated. A specific load was added over two slides of glass with 1 g of the formulation in between. This process was continued for about 1 min and the spreadability was calculated by measuring the spreading diameter [62].

### 4.9. In Vitro Release from NE-Hydrogel Formulation

The same method described in Section 2.6 was adopted to determine the percentage of FA released from the FA-NE-hydrogel formulation compared to optimized FA-NE. 

### 4.10. Kinetic Study

To demonstrate the mechanism by which the drug would be released from the developed formulations, a kinetic study was carried out. Different kinetic models were applied to determine the mechanism of release and the correlation coefficient (R^2^). The best-fit model would be obtained when a linear plot and the maximum value of R^2^ were achieved [24]. The kinetic models, represented by a graph that showed drug concentrations against time (T), were applied in this study, including zero-order, first-order, Higuchi, and Korsmeyer–Peppas kinetic models [59]. 

### 4.11. Stability Test

According to the International Conference on Harmonization (ICH) guidelines, the developed FA-NE-hydrogel was investigated for its stability over 3 months of storage. The sample was stored in a stoppered container and kept in 2 different conditions: room temperature (25 ± 2 °C/60 ± 5% RH) and refrigeration (4 ± 3 °C). The stored sample was evaluated for certain characteristics such as pH, viscosity, spreadability, and in vitro release [63]. 

### 4.12. Animals

#### 4.12.1. Animal Handling

Male Wistar rats supplied from the Experimental Animal Research Centre at King Saud University, Riyadh, KSA, with an average weight of 220–250 g, were used for in vivo studies. The animals were maintained in the animal house (12 h light/dark cycles) at ambient temperature and kept on standard laboratory food and drink during the whole study. 

#### 4.12.2. Declaration of Ethical Approval

All animal handling and experiments were conducted in accordance with the guidelines of ethical conduct for animal use at King Faisal University. In addition, the protocol of this study was issued by the Research Ethics Committee (REC) of King Faisal University, approval number KFU-REC/2022-May–ETHICS17.

### 4.13. In Vivo Study

#### Skin Irritation Test

It is very essential to perform a skin irritation test in order to assure the safety of a topical preparation when applied to the affected area over the skin. Male Wistar rats were prepared for this study by shaving the hair from their back using digital clippers. FA-NE-hydrogel was consistently applied over the shaved area and the skin was observed for any reaction for 7 days. To evaluate the obtained reactions, a score of 0, 1, 2, or 3 would be applied, referring to no reaction and slight, moderate, and severe erythema with or without edema, respectively [64].

### 4.14. Antibacterial Study

Representative strains for Gram-positive and Gram-negative bacteria were utilized to assess the antibacterial behavior of the studied formulations. The bacterial strains were supplied from the American Type Culture Collection (ATCC), namely *Staphylococcus aureus* (ATCC 29213) as an example of Gram-positive bacteria and Escherichia coli (*E-coli*) (ATCC 25922) as an example of Gram-negative bacteria. This study was carried out using Moller Hinton Agar, the media for the bacteria to be cultured, distributed in Petri dishes. Three wells of 6 mm diameter were prepared in each dish to which the examined formulation was packed. The examined formulations were FA-NE-hydrogel, blank NE-hydrogel, and FA suspension. The prepared Petri dish was incubated for 24 h at 37 ± 1 °C. The diameter of the observed inhibition zone was measured, which considered signs of antibacterial activity of the formulation. Each investigation was performed at least three times with the mean value ± SD. 

### 4.15. Morphology of Bacterial Cells Treated with FA-NE-Hydrogel Formulation

The morphology of the bacterial strains following treatment with the FA-NE-hydrogel formulation was assessed using scanning electron microscopy (SEM): 100 μg of FA-NE-hydrogel was mixed with 100 μL of Mueller–Hinton broth and 10 μL of microbe (about 1.5 × 10^6^ CFU/mL), and then incubated for 1 h. Afterwards, the mixture was centrifuged for 20 min at 15,000 rpm, removing the supernatant, and suspending the pellets in a normal saline. Then, 50 μL of the suspension was dispensed over the slide and left to dry. Then, the sample was fixed in 3% glutaraldehyde for 3 h and examined for its morphology using a scanning electron microscope. SEM imaging was adopted for the untreated bacteria to be a reference control [10]. The morphology of the treated and untreated bacteria was investigated at 30 kv.

### 4.16. Statistical Analysis

The results are expressed as the mean ± standard deviation (SD). If *p*-value < 0.05, a significant difference would be detected. Student’s *t*-test was performed to identify the statistical differences between the groups. All statistical analysis was confirmed by SPSS statistics software, version 9 (IBM Corporation, Armonk, NY, USA). One-way analysis of variance (ANOVA) was implemented using Design-Expert, version 12.0 (Stat-Ease, Minneapolis, MN, USA). 

## Figures and Tables

**Table 1:** Values of all factors and their perceived responses for different formulated FA-NE preparations.

Formula	Factors Values			Response Values		PDI
	A (%)	B (%)	C (%)	R_1_ (nm)	R_2_ (%)	
F1	20	5	5	257 ± 4.5	69 ± 3.0	0.355 ± 0.004
F2	20	10	5	234 ± 3.6	77 ± 2.6	0.333 ± 0.02
F3	15	10	7.5	209 ± 2.6	89 ± 3.6	0.237 ± 0.022
F4	15	7.5	10	213 ± 2.2	85 ± 3.1	0.242 ± 0.018
F5	25	5	7.5	308 ± 4.8	58 ± 2.5	0.402 ± 0.011
F6	20	7.5	7.5	247 ± 3.6	74 ± 2.3	0.318 ± 0.016
F7	25	7.5	5	291 ± 3.0	60 ± 2.6	0.321 ± 0.009
F8	20	10	10	228 ± 2.4	79 ± 3.4	0.345 ± 0.022
F9	15	5	7.5	223 ± 2.9	80 ± 3.5	0.362 ± 0.008
F10	25	7.5	10	278 ± 2.8	63 ± 2.6	0.355 ± 0.013
F11	25	10	7.5	269 ± 2.2	66 ± 3.2	0.380 ± 0.016
F12	20	7.5	7.5	244 ± 3.6	76 ± 3.6	0.403 ± 0.015
F13	15	7.5	5	219 ± 3.0	83 ± 4.1	0.396 ± 0.012
F14	20	7.5	7.5	246 ± 3.3	75 ± 3.2	0.375 ± 0.019
F15	20	5	10	251 ± 3.5	71 ± 3.5	0.348 ± 0.046

A: CEO concentration; B: Tween 80 concentration; C: Transcutol^®^ P concentration R_1_: particle size (nm) and R_2_: in vitro release (%).

**Table 2:** Statistical analysis of responses.

Source	R_1_		R_2_	
	F-Value	*p*-Value	F-Value	*p*-Value
Model	260.50	<0.0001 *	288.66	<0.0001 *
A	1994.75	<0.0001 *	2250.00	<0.0001 *
B	245.84	<0.0001 *	302.50	<0.0001 *
C	24.11	0.0044 *	22.50	0.0051 *
AB	31.35	0.0025 *	0.5556	0.4896
AC	2.46	0.1777	0.5556	0.4896
BC	0.0000	1.0000	0.0000	1.0000
A^2^	38.06	0.0016 *	18.46	0.0077 *
B^2^	0.2521	0.6369	0.5128	0.5060
C^2^	4.94	0.0768	4.62	0.0844
Lack of Fit	2.89	0.2673	0.0833	0.9630

A: CEO concentration; B: Tween 80 concentration; C: Transcutol^®^ P concentration R_1_: particle size (nm) and R_2_: in vitro release (%); *, significant.

**Table 3:** Fit statistic data for all responses.

R^2^ Analysis	R_1_	R_2_
R^2^	0.9979	0.9981
Adjusted R^2^	0.9940	0.9946
Predicted R^2^	0.9714	0.9927
Adequate Precision	52.2577	56.1416
Model		
Remark	Quadratic	Quadratic

**Table 4:** Adjusted constrains for selected factors and observed response predicted against observed value of the optimized FA-NE formulation.

Selected Factors	Symbol	Constrains
CEO concentration	A	In range
Tween 80 concentration	B	In range
Transcutol^®^ P concentration	C	In range
Observed response		
Particle size (nm)	R_1_	Minimize
In vitro release (%)	R_2_	Maximize
Observed response	Predicted values	Observed values
R_1_ (nm)	208.13 ± 2.23	202.4 ± 3.55
R_2_ (%)	89.03 ± 0.67	90.66 ± 3.43

**Table 5:** Drug release kinetics from investigated formulations.

Formulation	R^2^ Value			
	Zero Order Kinetic	First Order Kinetic	Higuchi Kinetic	Korsmeyer-Peppas Kinetic
Optimized FA-NE	0.8147	0.4314	0.9772	0.9717
FA-NE-hydrogel	0.9461	0.5477	0.9843	0.9741

**Table 6:** Antibacterial activity of examined formulations against different bacterial strains.

Bacterial Strain	Inhibition Zone (cm)		
	FA-NE-Hydrogel (a)	Blank NE (b)	Marketed FA (c)
*Staphylococcus aureus*	4.43 ± 0.12 *#	1.97 ± 0.15 *	4.11 ± 0.11 #
*E-coli*	2.40 ± 0.11 *#	2.10 ± 0.10 *	Negative

Values are expressed as mean ± SD. * (*p* < 0.05) compared to marketed FA; and # (*p* < 0.05) compared to blank NE formulation.

**Table 7:** BBD data of the selected factors along with their level of variations.

Independent Variable	Symbol	Level of Variation		
		Lowest (−1)	Central (0)	Highest (+1)
CEO concentration (%)	A	15	20	25
Tween 80 concentration (%)	B	5	7.5	10
Transcutol ^®^ P concentration (%)	C	5	7.5	10

## Data Availability

The raw data supporting the conclusions of this article will be made available by the authors on request.

## Supplementary Materials

The following supporting information can be downloaded at: https://www.mdpi.com/article/10.3390/gels10040268/s1, Figure S1: Inhibition zone diameter caused by investigated formulations; (a) FA-NE-hydrogel, (b) blank NE-hydrogel and (c) marketed FA on different bacteria; (A) *Staphylococcus aureus*, and (B) *E-coli*.
