# Supplementary material for: Enhancing the Topical Antibacterial Activity of Fusidic Acid via Embedding into Cinnamon Oil Nano-Lipid Carrier
Source: Gels. 2024 Apr 16;10(4):268. doi: 10.3390/gels10040268 (PMC11049292; doi:10.3390/gels10040268)
Supplement: Supplementary file 1 [file gels-10-00268-s001.zip › gels-2924905-supplementary.pdf]

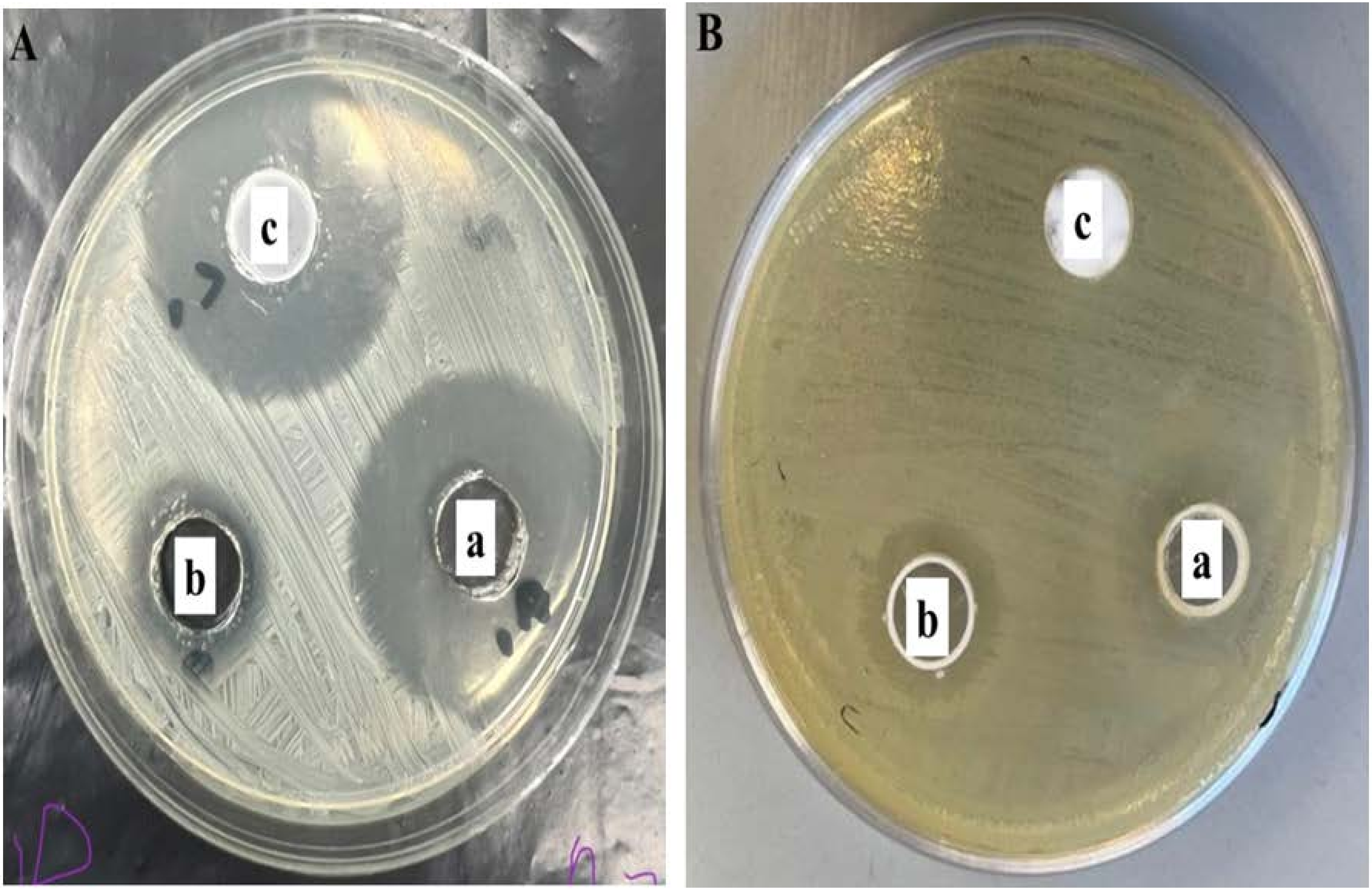

Figure S1. Inhibition zone diameter caused by investigated formulations; a) FA-NE-hydrogel, b) blank NE-hydrogel and c) marketed FA on different bacteria; A) Staphylococcus aureus, and B) E-coli.
